# Deciphering the role of tau in neurodegeneration using Adeno-Associated Viral (AAV) vectors to express human tau in the mouse forebrain

**DOI:** 10.1186/1750-1326-8-S1-P27

**Published:** 2013-09-13

**Authors:** Aurélien Lathuilière, Stéphanie Papin, Matthias Cacquevel, Paolo Paganetti, Andreas Muhs, Graham Knott, Bernard Schneider, Patrick Aebischer

**Affiliations:** 1 Brain Mind Institute/Ecole Polytechnigue Fédérale de Lausanne (EPFL), Lausanne, Switzerland; 2 AC Immune SA, Lausanne, Switzerland; 3 Centre of Interdisciplinary Electron Microscopy/Ecole Polytechnigue Fédérale de Lausanne (EPFL), Lausanne, Switzerland

## Background

Tau related pathology is the most reliable predictor of cognitive decline in Alzheimer’s disease. However, it remains unclear by which mechanisms tau contributes to neurodegeneration and neuronal dysfunction. The use of viral vectors provides an effective approach to replicate cardinal features of tauopathies in the mouse brain. AAV vectors were designed for overexpression of various forms of human tau, in order to dissect the mechanisms underlying tau hyperphosphorylation, aggregation and neurotoxicity *in vivo.*

## Materials and methods

We generated AAV constructs encoding various forms of the human tau protein, including wild-type (WT) tau, a mutant form causing frontotemporal dementia (P301S) and a tau variant previously reported as aggregation-deficient (I277P/ I308P). To induce transgene expression mainly in the forebrain, AAV6 vectors were bilaterally injected in the lateral ventricles (ICV) of mouse neonates. The injected mice were analyzed at various time points to compare the kinetic of the pathology induced by these different forms of tau. Using histological, biochemical and ultrastructural analysis, we assessed the deposition of phosphorylated and aggregated forms of tau, as well as the induced pathology. Resulting changes in animal behavioral performances were determined using specific tests.

## Results

AAV-injected mice displayed a somatodendritic and axonal accumulation of human tau protein in various brain regions. Pathologic forms of tau, both hyperphosphorylated and misfolded, were found to accumulate as soon as several weeks post-vector injection. A significant neurodegeneration was also observed in regions where tau was expressed at highest level. In addition, mice injected with WT and P301S tau-expressing vectors displayed a progressive decline in motor performance, most severe in the P301S mutant. In contrast, the overexpression of the I277P/I308P tau variant did not induce any detectable motor phenotype. Strikingly, the I277P/I308P tau variant was clearly less hyperphosphorylated than WT tau, and was associated with cytoskeletal changes in the axonal and dendritic compartments. Interestingly, the animal motor performance of WT and I277P/I308P tau expressing animals was significantly correlated with the abundance of specific tau phospho-epitopes.

## Conclusion

Overall, ICV delivery of tau-expressing AAV vectors in mouse neonates can efficiently induce robust neuronal tau pathology in the adult forebrain. The resulting accumulation of human tau leads to aggregation, hyperphosphorylation and behavioral defects, including for the WT form of tau implicated in Alzheimer’s disease. By allowing side-by-side comparison of different tau variants *in vivo*, our approach provides novel insights in the pathological processes mediated by tau and leading to neuronal dysfunction and death.

